# Ergothioneine, Ovothiol A, and Selenoneine—Histidine-Derived, Biologically Significant, Trace Global Alkaloids

**DOI:** 10.3390/molecules27092673

**Published:** 2022-04-21

**Authors:** Geoffrey A. Cordell, Sujeewa N. S. Lamahewage

**Affiliations:** 1 Natural Products Inc., Evanston, IL 60202, USA; 2 Department of Pharmaceutics, College of Pharmacy, University of Florida, Gainesville, FL 32610, USA; 3 Department of Chemistry, Iowa State University, Ames, IA 50011, USA; sujeewa@chem.ruh.ac.lk; 4 Department of Chemistry, University of Ruhuna, Matara 81000, Sri Lanka

**Keywords:** ergothioneine, ovothiol A, selenoneine, chemistry, biology, biosynthesis, distribution

## Abstract

The history, chemistry, biology, and biosynthesis of the globally occurring histidine-derived alkaloids ergothioneine (**10**), ovothiol A (**11**), and selenoneine (**12**) are reviewed comparatively and their significance to human well-being is discussed.

## 1. Introduction

**Figure 1:**
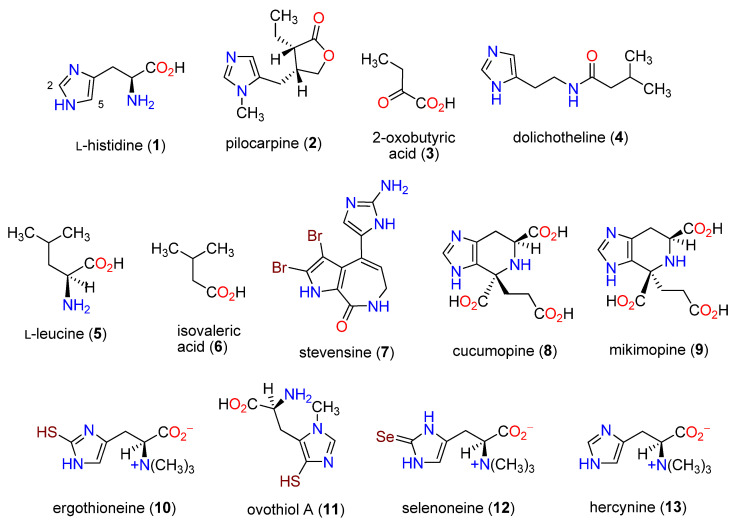
Representative histidine-derived alkaloids.

Histidine-derived (**1**) alkaloids are rare in nature, and when **1** does serve as a precursor fragment, the products are invariably biologically and biosynthetically interesting, as illustrated in Figure 1. The most important plant-originating alkaloid derived from histidine (**1**) is pilocarpine (**2**), isolated from “jaborandi”, the leaves of *Pilocarpus jaborandi* Holmes (Rutaceae), native to Brasil [1]. It acts as a muscarinic agonist and is used as an antidote to atropine, for the treatment of glaucoma, and as a sialogogue. The biosynthetic origin of the scaffold beyond **1** remains to be established, with 2-oxobutyric acid (**3**) a biogenetic possibility in combination with a deaminated and oxidized histidine (**1**) side-chain [2]. Dolichotheline (**4**) originates from the cactus *Dolichothele sphaerica* Britton and Rose (Cactaceae) indigenous to Texas [3,4]. In biosynthetic studies, histidine (**1**) was the primary precursor, and the additional carbon atoms originated from L-leucine (**5**) following conversion to isovaleric acid (**6**) [5]. Cell cultures of the marine sponge *Axinella corrugata* (formerly *Teichaxinella morchella*) showed [6] that histidine (**1**), ornithine, and proline were precursors of the alkaloid stevensine (**7**) [7].

Opines are compounds present in plant crown gall or hairy root tumors produced by *Agrobacterium* and *Rhizobium* pathogenic bacteria, respectively. Two recent isolates with **1**-derived fragments are cucumopine (**8**) from strains of *A. tumefaciens* and *A. rhizogenes* [8] and mikimopine (**9**) from hairy root cultures of *A. rhizogenes* NIAES 1724 [9,10]. These alkaloids are diastereomers at the C-4 position, as 4*R*,6*S* for **9** and 4*S*,6*S* for **8**, respectively [10].

This review brings focus to an intriguing group of histidine-derived alkaloids in which sulfur was inserted at either the C-2 position on the imidazole ring, e.g., ergothioneine (**10**), or at the C-5 position, e.g., ovothiol A (**11**), or by selenium at the C-2 position, e.g., selenoneine (**12**). Methyl groups are found at the histidine N_a_ or N-3 positions. As is be illustrated, their structural similarities belie their distinctive biosynthetic pathways.

## 2. Ergothioneine (10)

### 2.1. Occurrence

The oldest of the sulfur-containing alkaloids is ergothioneine. In 1909, Tanret isolated an alkaloid containing sulfur from ergot, *Claviceps purpurea* Tul. (Clavicipitaceae), established a molecular formula, C_9_H_15_N_3_S, and named the isolate ergothioneine [11]. Barger and Ewins, two years later, proposed the structure of a betaine, **10**, also from ergot, based on reactivity and degradation reactions [12]. Subsequently, standardization issues related to the accurate determination of uric acid in pigs’ blood were traced to an interfering compound labeled “substance X”, which was isolated and explored chemically [13]. The name of the isolate was later changed to “sympectothion”; although no structure was proposed [14]. Other researchers pursuing the same analytical issue isolated a sulfur-containing alkaloid from corpuscles and named it thiasine [15]. Surprisingly, the person who had purified the metabolite in crystalline form five years earlier, Helen David Dugdale, was not a co-author. Subsequently, the same group identified the structure of “thiasine” to be the same as that of **10** from ergot based on degradation, color reactions, and direct comparison [16,17], and through correlation of reactions, deduced that “sympectothion” was also the same alkaloid. Although a name change to “thioneine” was suggested [17], the studies that followed have respectfully used the original name proposed by Tanret, ergothioneine.

The alkaloid gives a characteristic red color with *p*-diazobenzenesulfonate in the presence of NaOH [12] through a reaction that was later developed as a highly specific, quantitative test [18]. Analysis of various animal blood samples showed that pig blood had the highest level of **10** (~2–5 mg/mL), whereas, in the blood of cattle, sheep, rabbits, dogs, and cats, the level of **10** was significantly lower (0.02–0.09 mg/mL) and paralleled the range of 0.03–0.08 mg/mL found in humans [18]. More recently, the biosynthetic precursor of **10**, hercynine (**13**), was also detected in human biological specimens, including saliva and urine, with the highest concentration in whole blood (1.3 μM/L) [19]. The level of **13** was markedly below that of **10** (~66 μM/L) in whole blood, a determination of physiological significance. High levels of **10** have been associated with patients diagnosed with autoimmune disorders, such as rheumatoid arthritis [20] and Crohn’s disease [21].

In the early research phase, it was determined that the **10** detected in mammals originated in the diet [22]. An analysis of the human diet in various European countries for the ingestion of **10** indicated that the Italian diet had the highest levels of **10** for both adults and children [23]. Another early study found **10** in oats (*Avena sativa* L. (Poaceae)) [24], although the overall distribution of **10** in foodstuffs is now established as very limited [25]. Analytical studies revealed the highest dietary levels of **10** to be in mushrooms, particularly *Pleurotus osteratus* (oyster), *Lentinula edodes* (shiitake), and *Grifola frondosa* (maitake) [26]. In a further analysis of foodstuffs, high levels of **10** were found in porcini (*Boletus edulis*) (526 mg/kg) and oyster mushrooms, and lower levels in chicken liver (10.76 mg/kg), as well as pork liver and kidney, black beans, red kidney beans, and garlic (3.11 mg/kg) [25]. All other foods tested had very low or non-detectable levels of **10**. Subsequent studies confirmed the highest levels in porcini and yellow oyster mushrooms [27]. The important role of **10** in the American diet was discussed in the context of a “longevity vitamin” [28]. Reviews of the chemistry and biology of ergothioneine (**10**) are available [29,30,31,32].

For such an apparently simple and important alkaloid, it is surprising that there are few syntheses of ergothioneine (**10**) available [33,34,35], and only one of the L-(+)-isomer [36]. An improved synthesis was described more recently, affording **10** in 70% overall yield from *N*-benzyl protected histidine, and the route was used for the synthesis of ^2^H-labeled **13,** which was then transformed into labeled **10** [37]. There are no published syntheses of the (-)-isomer, a compound of biological interest.

### 2.2. Biology

The earliest study of ergothioneine (**10**) [12] indicated that “Like other betaines it has no marked physiological action.” The intervening years have found that not to be the case, and summaries of the mammalian distribution and biological effects of ergothioneine (**10**) are available [29,30,31,32,38,39,40,41]. Ergothioneine (**10**) appears at high concentrations in bone marrow, liver, kidney, erythrocytes, seminal fluid, and the eye lens and cornea [42,43,44,45,46]. The distribution of **10** occurs through a highly specific transporter encoded by *OCTN1* [46,47]. Evidence from Δ*OCTN1* mutants indicates that the encoded enzyme is the only mediator for the transportation of the **10** obtained from dietary sources [48,49].

Despite quite extensive studies, the precise biological targets of **10** are still not apparent. The most important role is regarded as an antioxidant [40], controlling reactive oxygen species (ROS) and reactive nitrogen species (RNS), such as peroxy, hydroxy, and peroxynitrite [50,51,52]. Because **10** can pass through the blood–brain barrier (BBB), the antioxidant activity may serve to provide protection from neurodegeneration [53,54,55]. NMDA-induced neurotoxicity was ameliorated by the intraperitoneal administration of ergothioneine (**10**) [56]. Ergothioneine (**10**) levels in whole blood were significantly lower in Singaporean patients over 60 years old, and those with only mild cognitive impairment possessed even lower levels, signaling low levels of **10** as a possible correlative risk factor for neurodegenerative disease [57]. In a longitudinal clinical study in Sweden of 3236 participants monitoring 112 plasma metabolites, higher levels of ergothioneine (**10**) were correlated with a health-conscious food pattern which lowered the risk of cardiometabolic disease and death [58,59].

### 2.3. Biosynthesis

Curiosity regarding the biosynthetic origin of ergothioneine (**10**) began in earnest in 1955. Eagles and Vars [22] had suggested in 1928 that the origin of **10** was 2-sulfanylhistidine (**14**), a metabolite from corn. In this biogenesis, the only other elaborating feature to reach **10** would be triple *N*-methylation. However, **14** was never detected in biological specimens [60,61] and did not enhance blood levels of **10** in rats [62,63]. Intestinal bacteria in chickens were also ruled out as a site of biosynthesis [61], and radiolabeled histidine and methionine (S- or methyl group labeled) did not produce labeled **10**, and it was concluded that the origin was dietary [63], based on feeding oats to rabbits and rats [64,65].

**Figure 2:**
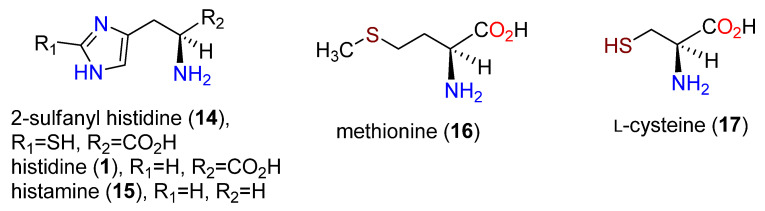
Structures of 2-sulfanylhistidine (**14**), histidine (**1**), histamine (**15**), methionine (**16**), and L-cysteine (**17**).

Ergothioneine (**10**) was detected in nine different fungi, where *Neurospora crassa* (85 mg/100 g dried cells) was the leading producer [66], while no production was detected in cultures in any of twelve diverse bacteria. The distribution of label from [2-^14^C]-acetate was the same in histidine (**1**) and **10**, inferring that **1** was a precursor requiring methylation and thiolation [67]. Labeled histamine (**15**) was not a precursor [68]. The intact incorporation of **1** into **10** in *C. purpurea* was established with **1** labeled in the imidazole ring and the side chain [69,70]. Parallel results with **1** were obtained by Melville et al. in *N. crassa* [71], who also showed that [^35^S]methionine (**16**) was incorporated into **10**; cysteine (**17**) was proposed as the source of sulfur (Figure 2). [*methyl*-^14^C]Methionine (**16**) was exclusively incorporated into the *N*-methyl groups. Based on the lack of both detection and incorporation of **14** into **10**, it was postulated that *N*-methylation preceded thiolation [71]. Labeling studies established that the two nitrogen atoms of **1** were retained in the biosynthesis of **10** and that all three *N*-methyl groups were derived from methionine and not from other potential single carbon sources [72].

The postulated first biosynthetic pathway step of triple *N*-methylation affords the alkaloid hercynine (**13**) [73], previously characterized by *Agaricus campestris* [74]. Ergothioneine (**10**) was found in *Mycobacterium tuberculosis* [75], and subsequently, **10** and **13** were quantitated in every *Mycobacterium* species examined [76], including at least six human strains and eleven non-human organisms. Notably, high levels of **10** were found in *M. kansasii*, *M. fortuitum*, *M. smegmatis*, and *M. avium*.

A detailed taxonomic study of the distribution of **10** and **13** determined that these metabolites were present in fungi in the Zygomycetes, Ascomycetes, Deuteromycetes, Myxomycetes, and Actinomycetales [77]. However, the biosynthesis of **10** was not observed in bacteria in the genera *Bacillus*, *Clostridium*, *Corynebacterium*, *Escherichia*, *Lactobacillus*, *Propionibacterium*, *Proteus*, *Pseudomonas*, *Staphylococcus*, *Streptococcus*, and *Vibrio*. True yeasts did not produce **10** or **13**, although some false yeasts in the Deuteromycetes did. The significance of their occurrence in the soil-based organisms in the Actinomycetales [77] supported the incorporation of **10** into plants through the roots [66] and rationalized the presence of **10** in the latex of *Hevea brasiliensis* (Willd. ex A. Juss.) Müll. Arg. (Euphorbiaceae) [78]. A trophic relationship was observed in the orchid *Gastrodia elata* Blume (Orchidaceae), which is dependent on the symbiotic fungus *Armillaria mellea*. The levels of **10** acquired by *G. elata* correlated with those in *A. mellea* [79]. In order to examine the organ-specific accumulation and the physiological effects of **10** in zebrafish, a reliable dietary source was needed [80]. Analysis of various options revealed oyster mushrooms, the alga *Arthrospira platensis* (spirulina), and the cyanobacterium *Oscillatoria* sp. to be leading sources, with other cyanobacteria also producing **10** and its precursor **13**. Indeed, cyanobacteria, such as spirulina, are regarded as a promising source of **10** for the future [80,81].

**Figure 3:**
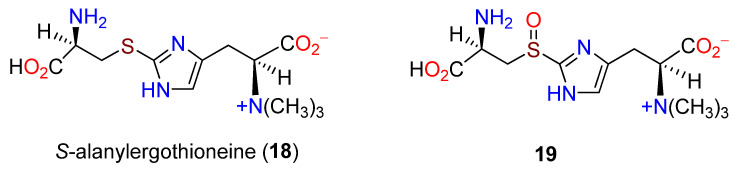
Structures of *S*-alanylergothioneine (**18**) and **19**.

Labeling studies with **1** and **16** indicated that the biosynthesis in mycobacteria (*M. tuberculosis* and *M. smegmatis*) paralleled the pathway in fungi [76], supported by the incorporation of **1** into **13**. Histidine (**1**) added to cultures of *M. smegmatis* enhanced the level of **13**, and the conversion of **13** to **10** was highly dependent on available sulfur. An intermediate between **13** and **10** [71] was proposed to be *S*-alanylergothioneine (**18**) [82], but this was not substantiated. A cell-free preparation from *N. crassa* converted **1** to **13** after 6 h [83], and a single methyltransferase was purified and established as responsible for all three *N*-methylation steps [84]. A sulfoxide derivative, *S*-(β-amino-β-carboxyethyl)ergothioneine sulfoxide (**19**), was characterized from *N. crassa* cultures supplied with [^14^C]hercynine (**13**) and **17** (Figure 3). The intermediate was converted to **10** by a pyridoxal-requiring enzyme [85].

### 2.4. Genetic Studies

**Figure 4:**
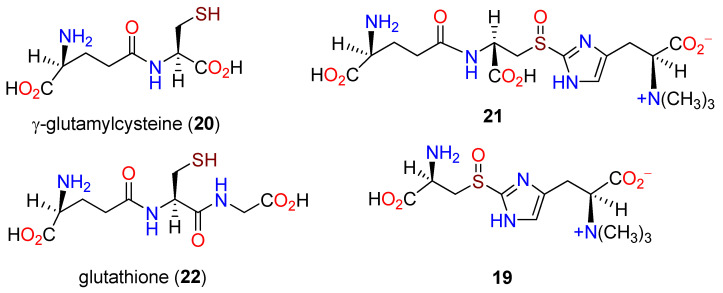
Structures of γ-glutamylcysteine (**20**), **21**, glutathione (**22**), and **19**.

It was 40 years before further progress on the biosynthesis of **10** was reported. Seventy-eight methyltransferases were identified in the genome of *M. smegmatis*. Through searching for one adjacent to a PLP-binding protein, a five gene cluster was identified, designated as *egtABCDE*, and shown to be responsible for the biosynthesis of **10** [86]. The suspected methyltransferase EgtD was cloned and expressed in *E. coli*, and L-histidine (**1**) and its *N*,*N*-dimethyl derivative were identified as the preferred substrates. EgtA, EgtC, and EgtD displayed sequence homology to a γ-glutamyl cysteine ligase [87], a class II glutamine amidotransferase, and a PLP-binding protein, respectively, leaving EgtB functionally unassigned with an *N*-terminal unit similar to a non-heme iron(II) motif. The capacity of EgtB to carry out oxidative desulfuration was evaluated with γ-glutamyl cysteine (**20**), which, in the presence of **13**, produced **21**. When EgtC was added, the sole product was the previously characterized sulfoxide **19**. Neither **1**, **17**, nor glutathione (**22**) were substrates for EgtB, implying that **21** was a requisite intermediate in *M. smegmatis* [86]. Culturing **13**, γ-glutamylcysteine (**20**), recombinant EgtB and EgtC, and a PLP-dependent β-lyase from *Erwinia tasmaniensis* afforded **10** exclusively (Figure 4).

EgtC, as an amidohydrolase, is a member of the superfamily of Ntn-hydrolases [88] which removes the glutamyl residue from the intermediate **20** to afford **19**. Crystallographic examination of EgtC with the substrate identified the binding sites and allowed the stereochemistry of the sulfoxide group to be determined as *S* [89]. This conclusion was also reached [90] based on the crystallographic examination of the complex of EgtB from *Mycobacterium thermoresistibile* with **13**, **20**, and Mn^2+^. EgtB is a non-heme iron enzyme, and the X-ray structure indicated that the two substrates were bound with three histidine residues at an octahedral iron-binding site, with the thiyl radical attacking C-2 on the imidazole ring to create the C-S bond [90].

EgtD is a methyltransferase which conducts the three successive *N*_a_-methylation reactions on L-histidine (**1**) to yield **13** [91]. Crystal structures of EgtD alone and with *N_a_*,*N_a_*-dimethylhistidine (**23**) and *S*-adenosylhomocysteine (**24**) were determined, which identified the active sites for substrate specificity, including for L-tryptophan (**25**). In addition, a bioinformatics search revealed the presence of EgtD homologs in fungal genomes as a frequent occurrence in both ascomycetous and basidomycetous fungi [87]. Other searches indicated that EgtB and EgtD homologs are present in a wide range of proteobacteria and cyanobacteria [92].

**Figure 5:**
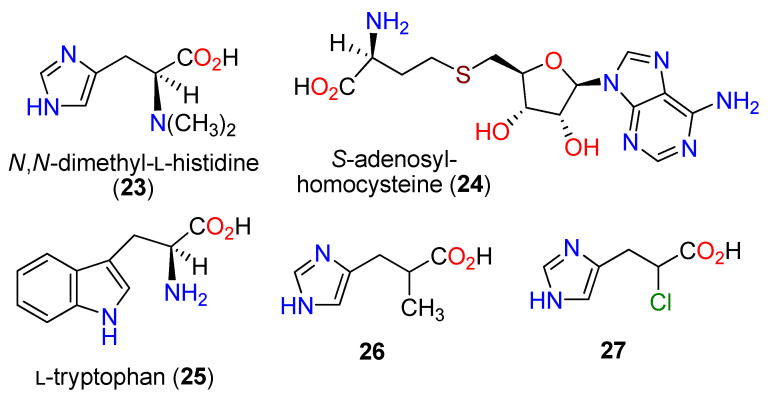
Structures of *N*,*N*-dimethyl-L-histidine (**23**), *S*-adenosylhomocysteine (**24**), L-tryptophan (**25**), **26**, and **27**.

Examination of the substrate binding in the methyltransferase EgtD from *M. smegmatis* for the conversion of **1** to **13** determined that the first methylation, conducted by a class I methyltransferase site [93], is rate-limiting as part of an ordered sequence [94]. In addition, the tri-*N*-methylated product of EgtD **13** is a competitive inhibitor since, when in place, it prevents the approach of the sulfonium center of methionine. Two amino acid residues, Asn166 and Gly161, are critical in the sequential methylation process, as the substrate for the final methylation step requires a re-orientation of the *N_a_*,*N_a_*-dimethylamino group to provide access to the nitrogen lone pair for methionine. Knowledge of the structural binding studies prompted the development of 3-(1*H*-imidazol-4-yl)isobutyric acid (26) and 3-(1*H*-imidazol-4-yl)-3-chloropropionic acid (27) as strong inhibitors of EgtD (Figure 5) [94]. In addition, when EgtD from *M. tuberculosis* was phosphorylated at Thr213 by the serine/threonine protein kinase PknD, the biosynthesis of **10** proliferated [95]. Interestingly, through an unknown mechanism, upregulation of the biosynthesis of 10 occurred in a gliotoxin-deficient strain of *Aspergillus fumigatus* [96] and a mycothiol-deficient strain of *M. smegmatis* [97].

The mycobacterial *egtB* gene encodes for the enzyme EgtB, which conducts the oxidative coupling of hercynine (**13**) and γ-glutamyl-cysteine (**20**) to form the sulfoxide **21**. Homologs of EgtB were characterized by the fungi *Collectotrichum graminicola* and *Neurospora crassa* [98], *Aspergillus fumigatus* [99], and the yeast *Schizosaccharomyces pombe* [100]. The levels of **10** were significantly higher in the conidia than in the mycelia [98]. When the gene encoding for the trimodular EgtA in *A. fumigatus* was deleted, the mutant was unable to produce **10**, showed reduced conidiation, and was more susceptible to stress from heavy metals and menadione [99]. The Δ*egt1* mutant from *S. pombe* did not produce **10** or its precursors, **13** and **21** [100], although the sulfoxide **21** accumulated in cultures of a Δ*egt2* mutant which was therefore encoding the enzyme responsible for conversion of the sulfoxide **21** to **10** [100].

**Figure 6:**
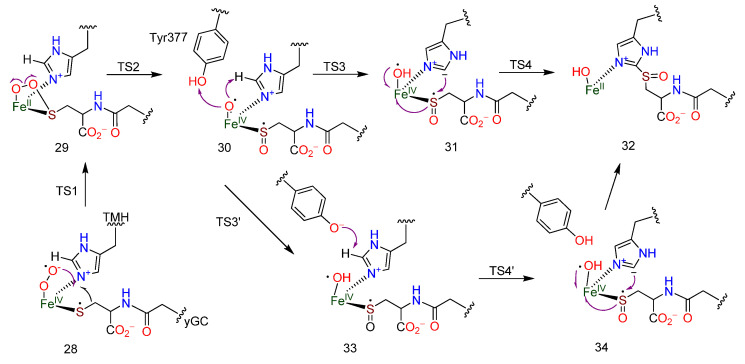
Postulated mechanism and transition states for EgtB based on QM/MM calculations (adapted from ref. [105]).

A priori, there are two steps in the overall reaction enacted by EgtB to reach **21**, regioselectively linking sulfur to the imidazole ring of **13** and oxidizing the sulfur to the sulfoxide; both mechanistic sequences were proposed based on the interpretation of ovothiol (**11**) biosynthesis (vide infra) [101,102]. Two other studies of the in silico assessment of the mechanism of EgtB were presented using quantum mechanics (QM) [103] and quantum mechanics/molecular mechanics (QM/MM) [104]. The proposed role of the critical Tyr377 residue and the rate-limiting steps resulting from the assessments were different; nucleophilic C-S bond formation in one instance [104] or the involvement of a sulfenic acid intermediate, with deprotonation as the rate-limiting step in the other case [103]. Through further QM/MM calculations and by examining the energy profile for alternative mechanisms, a new mechanistic proposal evolved [105]. Placing dioxygen in the structure of EgtB revealed two orientations (“side-on” and “end-on”) in relation to the Fe atom, of which the former was deemed not to be pathway relevant. It was proposed that after the Fe(IV)-S bond is formed in **28**, and dioxygen adds to create a radical anion, the planar Fe-O-O-S ring is formed with the **17** S radical as in **29**. Fragmentation of the O-O linkage forms the sulfoxide **30**, the Fe(IV)-O radical, which then abstracts a proton from C-2 to generate the imidazole anion in **31**. This was deduced to be the rate-limiting step, with an energy barrier of 21.7 kcal/mol. The imidazole anion then attacks the sulfoxide displacing Fe(IV) and creating the intermediate **32** [105]. The importance of the Tyr377 residue was explained through an alternative pathway involving **33** and **34** in which the C-2 proton is abstracted by a tyrosyl radical generated through proton abstraction by the Fe-O radical (Figure 6) [38]. The quintet surface pathway for the initial reactant **28** had the lowest energy profile [105].

The crystal structure of the EgtB_Cth_ from *Candidatus Chloracidobacterium thermophilum* exhibited both EgtB- and Egt1-type activities [106] and was compared in detail with the EgtB_Mth_ from *M. thermoresistibile* [90]. The *egt* operon of *N. crassa* eliminates two biosynthetic steps compared with the pathway in mycobacteria [107]. Two questions were addressed, why does this EgtB exhibit this substrate flexibility, and can the system be adapted to enhance the receptivity for **17** as a substrate rather than **20**? The binding residues through iron for **13** between EgtB_Mth_ and EgtB_Cth_ were conserved as Gln137, Asn414, and Trp415 in EgtB_Mth_ and as Gln156, Asn414, and Phe415 in EgtB_Cth_. The residues binding γ-Gly-Cys (**20**) in the two enzymes were identical, involving the two Arg residues, Arg 87 and Arg89, in EgtB_Mth_ and Arg103 and Arg 106 in EgtB_Cth_, in which the **17** carboxy group is also involved. The overall binding site in EgtB_Cth_ was more open, and double mutation of two amino acids (Asp52 and Ala420) close to the active site significantly enhanced Egt1 activity when **17** was the substrate with **13** [106].

**Figure 7:**
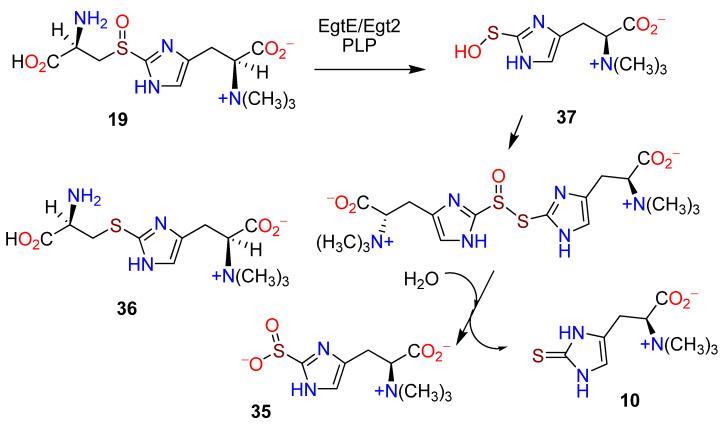
Incubation of EgtE with the thioether **36** and the sulfoxide **19** (adapted from ref. [108]).

**Figure 8:**
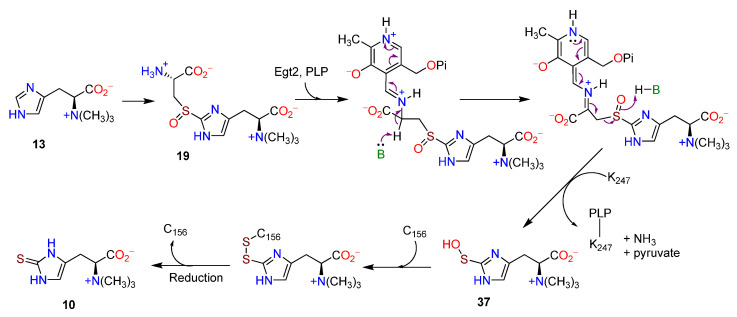
Postulated pathway from hercynine (**13**) to ergothioneine (**10**) involving PLP (adapted from ref. [38]).

EgtE is a PLP-dependent C-S lyase whose activity was confirmed in vitro [108,109]. The preferred utilization of the sulfoxide **19** by the recombinant EgtE from *M. smegmatis* was established through a postulated π-cation interaction [108]. A labile sulfenic acid intermediate **35**, proposed [108] as a part of the C-S lyase process in the biosynthesis of **10**, was confirmed through trapping and mass spectrometry [109]. C-S lyases usually act on thioethers [110], whereas in the biosynthesis of **10** the sulfoxide derivatives are substrates [108,111]. Incubation of EgtE with the thioether **36** and the sulfoxide **19** yielded different products. The thiol ether gave **10**, whereas the sulfoxide, in the absence of a reductant, gave **10** and the sulfenic acid derivative **35** in a 1:1 ratio (Figure 7) [108]. Based on the crystal structure of Egt2 from *N. crassa* with PLP covalently linked [109], a mechanistic model was proposed for the formation of **10** from **13** through **37** (Figure 8) [38]. The structural basis for the biosynthesis of **10** was summarized [112].

A BLAST search indicated that the genes for ergothioneine (**10)** biosynthesis were likely present in actinobacteria, pezizomycotina, cyanobacteria, basidomycota, bacteriodetes, and proteobacteria [86]. However, the physiological importance of this exceptionally broad distribution in nature remains to be discerned. Of 2509 prokaryote genomes surveyed for the five gene cluster for ergothioneine (**10**) biosynthesis, over 400 were deemed to have orthologs of *egtB* and *egtD*, some instances of which were considered to have occurred through horizontal gene transfer [110]. A parallel examination of more than 100 fungal genomes indicated a wide distribution of the *erg1* gene across all phyla, except for the Saccharomycotina subphylum.

### 2.5. Ergothioneine Production

Attempts at expanding the potential sources of **10** for production purposes have included studies in *E. coli* [113,114], *Aspergillus oryzae* [115], and the yeast *Saccharomyces cerevisiae* [116,117]. Through the heterologous transfer of two genes for ergothioneine biosynthesis from the maitake mushroom *Grifola frondosa* into *S. cerevisiae* E1118, and with daily additions of 1% glycerol for 7 days, the yield of **10** was 20.6 mg/L [117]. More successful were efforts in *S. cerevisiae* through carefully defining the parameters for the medium and potentiating the influence of added amino acids, transporters, and enzymes from other pathways for ergothioneine (**10**), leading to a yield of 598.8 mg/L [116]. The introduction of multiple copies of *egt1* from *N. crassa* into *A. oryzae* led to the accumulation of **13** [115]. When L-cysteine (**17**) was overproduced in *E. coli*, yields of **10** as high as 1.3 g/L were obtained with longer fermentation times [114].

## 3. Ovothiols

### 3.1. Occurrence

**Figure 9:**
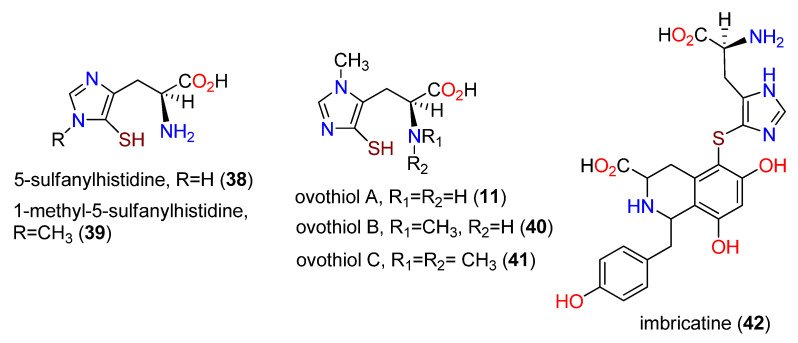
Structures of 5-sulfanylhistidine (**38**), 1-methyl-5-sulfanylhistidine (**39**), ovothiol A (**11**), ovothiol B (**40**), ovothiol C (**41**), and imbricatine (**42**).

Other thiohistidine derivatives include 5-sulfanylhistidine (**38**) as a constituent of adenochromine [118] and 1-methyl-5-sulfanylhistidine (**39**) isolated from the unfertilized eggs of the sea urchin, *Paracentrotus lividus* [119]. The eggs of a different sea urchin, *Strongylocentrotus purpuratus*, afforded the ovothiols A (**11**), B (**40**), and C (**41**), with increasing levels of side-chain *N_a_*-methylation [120,121]. Ovothiol A (**11**) is 3-methyl-5-sulfanyl-L-histidine and was subsequently obtained from the trypanosomatid *Crithidia fasciculata* [122] and *Leishmania donovani* [123]. The biological significance of ovothiol A (**11**), and its challenging isolation due to oxidative dimerization, led to the development of several total syntheses [124,125,126]. As the result of a total synthesis [124], the structure of a previously isolated alkaloid from *Paracentrotus lividus* was revised to that of ovothiol A (**11**). The ovalothiol A moiety is also apparent in the structure of the starfish alkaloid imbricatine (**42**), which has biosynthetically interesting *meta*-locations of the two phenolic groups on the tetrahydroisoquinoline ring [127,128], possibly implying a polyketide derivation for those aspects of the scaffold (Figure 9). These early studies on the ovothiols were reviewed [129], and some of the more recent studies were also summarized [38,130].

### 3.2. Biology

Ovothiols are free radical scavengers [121,131,132] and are noted for their antioxidant activity, which allows them to self-protect from various forms of oxidative stress [122,123,133,134]. The kinetic aspects of developing an antioxidant capacity were explored [135] since it provides protection for other organisms, particularly at critical life cycle stages, for example, during fertilization and larval development in the sea urchin [134], and for the mollusk, *Mytilus galloprovincialis*, during gametogenesis [136]. The ovothiols serve to defend against the host cell following parasite infection [137,138] and are distributed in the glandular cells and other tissues of marine Polychaeta, where they may function as signaling agents [139]. Ovothiol A (**11**) provides antioxidant activity in the lenses of fish [140], presumably being acquired through a dietary source. Oviothiol B (**40**) levels in *Skeletonema marinoi* are modulated by light [141], and **40** is present with several other antioxidants in the alga *Euglena gracilis* [142]. It also serves as a pheromone for marine worms and cone snails [143] and as an egg-release pheromone in Polychaeta [144]. Anti-inflammatory activity was observed in an in vitro system where endothelial dysfunction was induced by hypoglycemia [145], and **11** was effective against liver fibrosis in vivo [146,147]. Weak cytotoxicity was observed for **11** against the human liver carcinoma cell line Hep-G2 by inducing autophagy [148], and **11** also shows γ-glutamyl transpeptidase activity [149,150].

### 3.3. Biosynthesis

Intrinsically, only two biosynthetic processes are required to convert L-histidine (**1**) to ovothiol A (**11**), namely, regiospecific 3-methylation and regiospecific 5-sulfanylation. Based on the incorporation of ^35^S- and *methyl*-^14^C-labeled methionine and ^35^S-cysteine (**17**) in *Crithidia fasciculata*, it was concluded that sulfanylation preceded *N*-methylation [151]. A crude enzyme preparation requiring oxygen converted **1** to a 5-sulfanylated derivative **38** in the presence of cysteine, iron, and pyridoxal pyrophosphate (PLP). When PLP was not present, the intermediate *S*-(4′-l-histidyl)-l-cysteine sulfoxide (**21**) resulted, indicating that the C-S lyase requires PLP as the catalyst [152]. Thus, a fundamental difference was illuminated between the first steps in the formation of **10**, which involves triple *N-*methylation of the side chain amino group of **1** to **13**, and **11**, where the initial pathway step is 5-sulfanylation.

**Figure 10:**
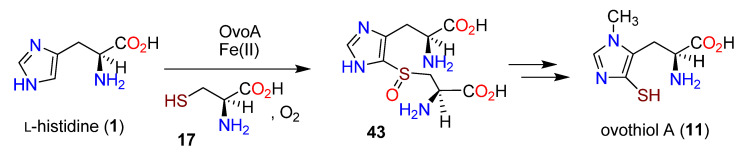
Formation of the sulfoxide intermediate **43** from L-histidine (**1**) and L-cysteine (**17**) by OvoA (adapted from ref. [101]).

The insertion of a sulfur atom onto an aromatic nucleus with the apparent requirement for both oxygen and iron implied a different enzyme mechanism in the instances of both **11** and **10**. A distinction was made between the EgtB, which inserts sulfur at C-2 in the biosynthesis of **10** and the postulated OvaA, which had a methyltransferase domain at the C-terminal [101]. An informatics search based on this distinction revealed 80 homologs of OvoA in proteobacteria, as well as in uni- and multicellular eukaryotes. Recombinant OvoA enzymes from *E. tasmaniensis* and *Trypanosoma cruzi* were generated, and the former was determined as the more active, with up to 140 turnovers per active site. Several thiol derivatives were explored as potential substrates, including γ-glutamylcysteine (**20**) and glutathione (**22**); L-cysteine (**17**) was strongly preferred. *N*-Methylated histidines were not accepted as substrates. OvoA, therefore, operates on L-histidine (**1**) and L-cysteine (**17**) to form the sulfoxide intermediate **43** (Figure 10) [101].

**Figure 11:**
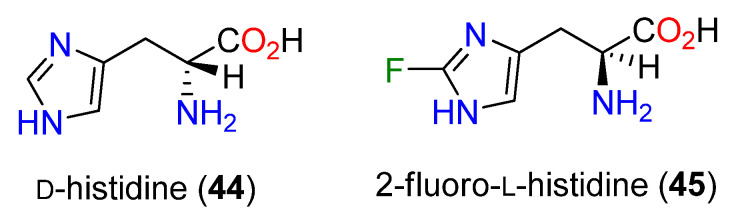
Structures of D-histidine (**44**) and 2-fluoro-L-histidine (**45**).

**Figure 12:**
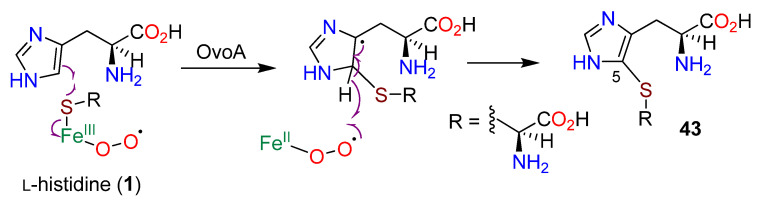
Reaction of L-cysteine (**17**) in the presence of Fe(III) (adapted from ref. [102]).

**Figure 13:**
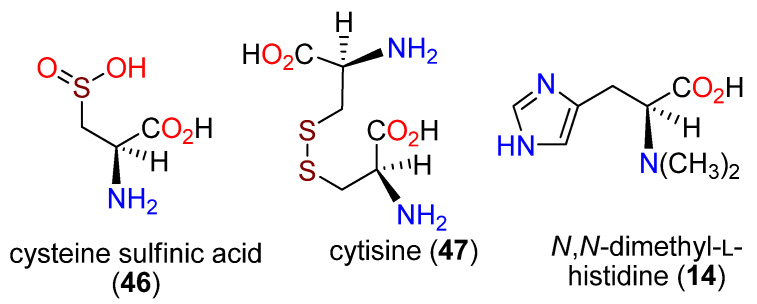
Structures of cysteine sulfinic acid (**46**), cytisine (**47**), and N,N-dimethyl-L-histidine (**14**).

Selective point mutations of an iron recognition area towards the N-terminal (His170, His 174, and Glu176) in each instance reduced OvoA activity 100-fold [101]. It was proposed that the initial step in the formation of **43** was the oxidation of L-cysteine (**17**) to an iron-bound sulfoxide, which is attacked by C-5 of **1**. Further study revealed that D-histidine (**44**), 2-fluoro-L-histidine (**45**), histidine amide, and histamine (**15**) could also serve as substrates and be 5-sulfanylated by OvoA (Figure 11) [102]. Assuming the mechanism is consistent, the observed utilization of the weakly nucleophilic **45** prompted a different mechanistic proposal (Figure 12) in which a Fe(III)-superoxide complex produces an L-cysteine thiyl radical and then attacks C-5 followed by aromatization in a manner analogous to a thiol-ene reaction [153]; sulfur oxidation occurs subsequently. OvoA also catalyzes three other reactions of L-cysteine (**17**): the oxidative coupling with hercynine (**13**) with the new bond generated at C-2 to afford **19**, and with either cysteine sulfinic acid (**46**) or cystine (**47**) (Figure 13) [154].

Further kinetic and ^2^H-labeling, as well as quantum mechanics studies in which the Y417 site was modulated with a 3′-hydroxytyrosine implant, suggested that C-S bond formation precedes the sulfur oxidation step conducted by OvoA [155]. The same modulated OvoA also demonstrated significantly higher (10% to 30%) dioxygenase activity [156]. In summary, OvoA and EgtB are responsible for the sulfoxide-generating processes in the biosynthesis of ovothiol A (**11**) and ergothioneine (**10**), respectively. Their substrates and reactants are quite different, however, and as a result, the regioselectivity of substitution on the imidazole scaffold is also different; OvoA uses **1** with **17** attacking the C-5 position, while EgtB catalyzes the reaction between **13** and **20** inserting the added fragment at C-2 [38].

When the substrate specificities of EgtB and OvoA were examined, there was a surprising outcome. EgtB did not utilize either of the two substrates of OvoA. On the other hand, OvoA demonstrated broader substrate specificity than EgtB, for it could also catalyze the reaction between hercynine (**13**) and L-cysteine (**17**) [107]. The product was **19,** in which substitution occurred at the C-2 position, not at C-5, effectively reorienting the original regioselectivity of the native substrates. OvoA could also catalyze the *N_a_*-methyl and the *N_a_*,*N_a_*-dimethylhistidines as substrates in reaction with **17**. However, in the former instance, a 2:3 mixture of 2-substituted and 5-substituted products were formed, whereas with *N_a_*,*N_a_*-dimethylhistidine (**14**), the dominant product was the 2-substituted regioisomer [107]. The binding pocket of OvoA, therefore, has the capacity, depending on the substitution of the *N_a_*-amino group of histidine, to flip the regioselectivity from C-5 to C-2. It is also important to recapitulate that the *direct* formation of **19** between **13** and **17** eliminates the two pathway steps conducted by EgtA and EgtC in the formation of **10** and therefore eliminates the inherent competition between the pathways of **10** and glutathione (**22**) for the common substrate [107]. Bioinformatic and biochemical analyses identified the fungus *Neurospora crassa* as displaying this more efficient biosynthetic pathway [157]. The gene *egt1* encodes for the non-heme iron enzyme Egt1 and uses γ-glutamylcysteine (**19**) and not L-cysteine (**17**) in reaction with **13**. However, the observed side reactions already mentioned and the reduced rate of the reaction makes this a non-viable approach for ergothioneine (**10**) production.

A Cytoscape network [158] for the EgtB domains revealed that fungal genes with this capacity were distinct from other bacterial clusters. One of these from *N. crassa* was of special interest. Biochemical information [159] indicated that deletion of *egt1* of *N. crassa* enhanced the sensitivity to oxidative stress, implying a loss of **10** formation [98]. The product of the cloned and expressed Egt1 with **13** and **17** was the sulfoxide **19** substituted at the C-2 position, together with a minor amount (12:1 ratio) of the cysteine sulfinic acid (**46**) [157]. The specificity of Egt1 for **17** compared with **19** was 62-fold. The important conclusion is that mycobacteria, and the fungus *N. crassa*, have different biosynthetic pathways to produce ergothioneine (**10**) involving the enzymes EgtA-EgtE in the former instance and Egt1-Egt2 in the latter [157].

### 3.4. Anaerobic Synthesis of Ergothioneine

More recently, it was disclosed that ergothioneine (**10**) is also produced under anaerobic conditions. Since in the aerobic pathway oxygen is central to the biosynthesis of the sulfoxide intermediate **19** (Figure 2), the requisite alternate mechanism became of interest. From the green bacterium *Chlorobium limicola* DSM 245, which functions under anoxic conditions, the homolog of EgtD was identified and designated as EanA [160]. The enzyme conducted the trimethylation of **1** to **13**, and in the genome, *eanA* was proximate to a gene encoding for EanB, a rhodanese-like sulfur transferase. This combination of genes was identified in the genomes of over 20 anaerobic bacteria and archaea. In the presence of the cysteine desulfurase IscS, EanB affected the transfer of sulfur to **13** to produce **10**, thereby identifying a third pathway for the biosynthesis of **10** [161]. The role of **10** in anaerobic organisms remains to be determined, given its established association with antioxidant activity and the removal of reactive oxygen species [50].

**Figure 14:**
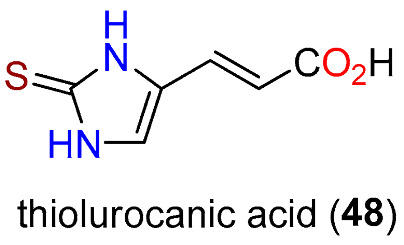
Structure of thiolurocanic acid (**48**).

However, the role of IscS has now been re-interpreted [162]. One of the features of the reaction on **13** with EanB in the presence of ergothionase [163], which converts **10** to thiol-urocanic acid (**48**) (Figure 14), was a precipitate that was analyzed predominantly for S_8_ accompanied by other polysulfides, as observed previously in the absence of a sulfur acceptor [164,165]. Incubation studies with S_8_ and dithiothreitol produced **10**, and the highest yield of **10** was obtained with potassium polysulfide (K_2_S_4_) [162]. Of the five cysteine residues in EanB only Cys412 was necessary for catalysis of the sulfur insertion reaction. Demonstration of this came through point mutation to a Ser residue which gave an inactive EanB, whereas a mutant in which the four other Cys residues were changed to Ala still produced **10**. The Cys412 active site is buried at the end of a 13 Å tunnel which supports the concept that the IscS-Cys328 complex could not reach the site, whereas a slim polysulfide could.

**Figure 15:**
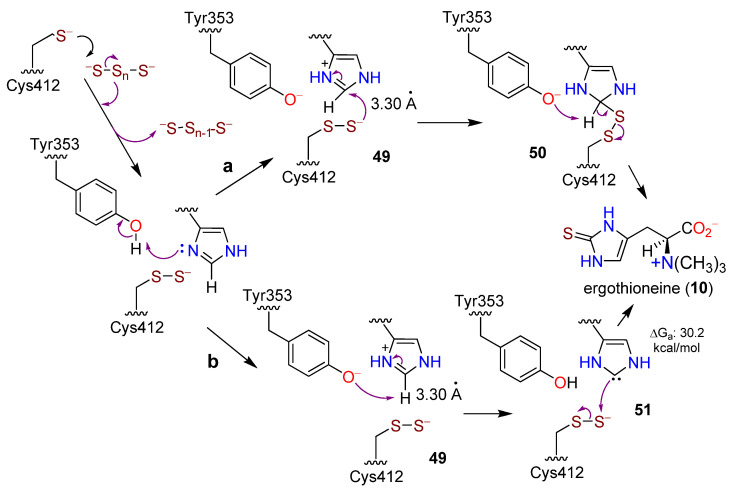
Proposed alternative mechanistic pathways a and b for the interaction of hercynine (**13**) and polysulfur in the presence of EanB to form ergothioneine (**10**) (adapted from ref. [162]).

Co-crystallization of EanB with polysulfide and **13** revealed a binary complex with the Cys persulfide in which the terminal sulfur atom of the Cys persulfide was only 3.2 Å from the imidazole C-2 [162]. Additionally, the Tyr353 residue in EanB was only 3.1 Å from the imidazole C-2. When Tyr353 was mutated, **10** formation was abrogated. QM/MM studies then suggested that Tyr353 provides the proton at N-1 of the imidazole unit in **13,** leading to an intermediate **49**. This was supported by free energy calculations in which Thr414 specifically assists in orienting **13** since mutation at the site also abrogated the production of **10**. After **49**, the Cys412 persulfide attacks at C-2 to form a tetrahedral intermediate **50**. Two different pathways were proposed for the return of the proton from **50** to Tyr353, one of which was favored energetically (Figure 15a). An alternative route from **49** removes the hydrogen from C-2, leaving a carbene intermediate **51,** which then attacks the terminal S atom of the polysulfide generating the thione at C-2 of **10** (Figure 15b) [162]. This was the first, and thus far only, reported instance of polysulfide as an unambiguous direct source of sulfur in biosynthesis. Other biosynthetic pathways also proposed a carbene intermediate [166,167].

**Figure 16:**
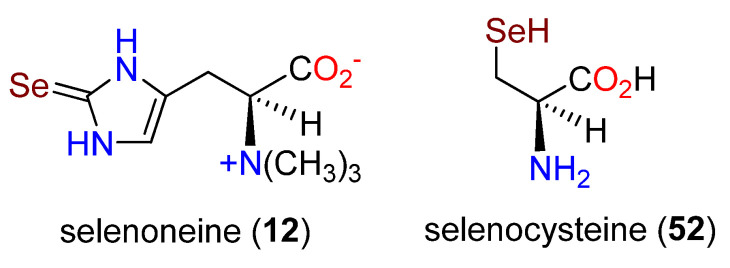
Structures of selenoneine (**12**) and selenocysteine (**52**).

Additional studies supported the intermediacy of an imidazole carbene [168]. In the presence of EanB and D_2_O, the C-2 proton in **13** is readily exchanged, and when Tyr353 was modified with the 3′,5′-difluoro analog, the exchange rate was increased 10-fold. It was proposed that Tyr353 acts as a Lewis acid in the activation of **13** and as a Lewis base in the deprotonation step at C-2. An attempt to prepare selenoneine (**12**) (Figure 16) (vide infra) using EanB, where the substitution at C-2 is by selenium rather than sulfur, was unsuccessful [168]. However, this alkaloid could be produced by *Schizosaccharomyces pombe* after the addition of sodium selenate [100,169], and EgtB_Cth_ can use selenocysteine (**52**) to produce the corresponding selenoxide from **13** [170].

### 3.5. Final Pathway Steps

**Figure 17:**
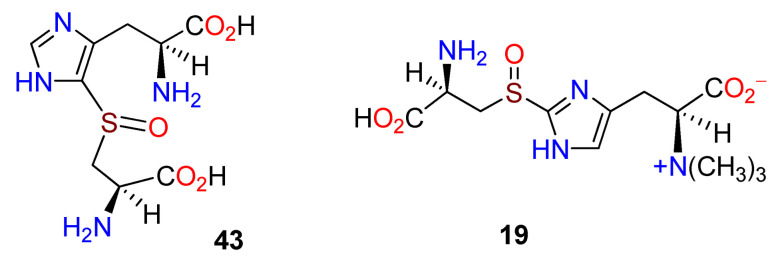
Structures of **43** and **19**.

Attention was also focused on the final pathway steps, the formation of **11** from **43,** which involves regioselective *N*-methylation at N-3 and sulfoxide reduction. Bioinformatics identified a gene in *E. tasmaniensis* classified as an ergothioneine C-S lyase [86], even though the organism does not produce **10**. When purified, the enzyme showed modest efficiency for the conversion of the cysteinyl-histidine sulfoxide derivative **19** and high efficiency for the conversion of **43** to 5-sulfanylhistidine (**38**), together with ammonia and pyruvate (1:1:1). The enzyme was designated as OvoB, and the crystal structure was used to examine the docking interactions of the substrates **43** and **19** (Figure 17) with both OvoB [171] and Egt2, the ergothioneine C-S lyase [109,172]. The observed interactions provided a rationalization for the substrate selectivity of the respective enzymes, Egt2 in the biosynthesis of **10** and OvoB in the formation of **11**.

### 3.6. Additional Sources

The search for an imidazole *N*-methyltransferase revealed 52 such enzymes in *E. tasmaniensis*, none of which was associated with the *Ovo* operon. However, a methyltransferase was one of the three domains in OvoA from *T. cruzi* [101] and was suggested to conduct the imidazole *N*-methylation [92]. When OvoA was cultured with **38** and SAM, the exclusive product was ovothiol A (**11**) through N-3 methylation [171].

Unicellular diatoms are microalgae found in the moist soils, waterways, and oceans and comprise a significant proportion of Earth’s biomass. They contribute to the ecosystem by producing much of the oxygen generated each year. OvoA homologs were identified in diatoms [101,173], and OvoA was indicated to be present in both centric and pennate diatoms [174]. From cultures of *Skeletonema marinoi*, a thiol fraction was identified that contained ovothiol B (**40**) [174], whose only previous isolation at the time was from the scallop *Chlamys hastata* [121].

The breadth of ovothiol distribution in nature is still being explored and continuously expanded based on searching for *ovoA*. A study of arthropods revealed a very surprising situation [175]. It was established that metazoans in the Porifera, Cnidaria, Echinodermata, and Hemicordata all carried the *ovoA* gene [130], whereas insects and fish do not carry the gene. A search relating to the Crustacea subphylum indicated at least eight insects (6 Hemiptera and 2 Diptera) had OvoA transcripts [175]. Marine arthropod studies indicated two clades for the OvoA sequences. One clade included twelve sequences from the copepods and three from the decapods, while the second clade had seven copepods, four decapods, and two amphipods. The expression of OvoA in generating an antioxidant was enhanced in the copepod *Calanus finmarchicus* when subjected to a toxic algal diet and when going through specific molting stages [175].

Bioinformatics analysis revealed the global distribution of OvoA and OvoB in aerobic Proteobacteria in numerous pelagic (open ocean) and other environments with high oxygen levels, such as surface and subsurface waters [176]. About 2% of the species were from deep-sea hydrothermal vents, and two species were known to be anaerobic [177,178]. This implies a possible alternative biosynthetic pathway for **11**, as observed for ergothioneine (**10**) biosynthesis in *Chlorobium limicola* [179]. In analyzing the bacterial OvoA proteins, two other aspects were revealed. The first was that some of the OvoA-like proteins lacked a methyltransferase domain, and secondly, the N-terminal of OvoB may be fused with OvoA, as observed in hydrozoans [173]. About 36% of the bacteria with *ovoA* and *ovoB* genes are regarded as human or animal parasites, with the inherent antioxidant activity possibly to protect the host from other organisms or to protect themselves from the immune response of the host [175].

## 4. Selenoneine

### 4.1. Introduction

**Figure 18:**
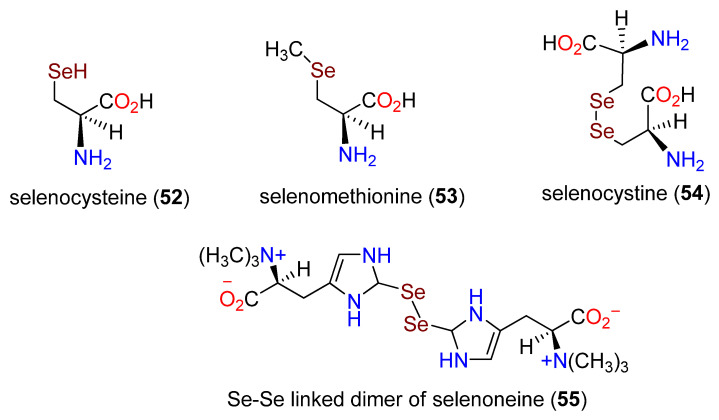
Structures of selenocysteine (**52**), selenomethionine (**53**), selenocystine (**54**), and the Se-Se-linked dimer of selenoneine (**55**).

One of the most interesting and biologically significant human alkaloids to be discovered in the past few years is selenoneine (**12**), which at physiological pH exists in the selenol form [180,181]. Selenium (Se) is an essential element for human health [182,183] and is present in a wide variety of foods, including some beans, nuts, meats, and soy products [184,185,186,187,188]. The recommended dietary intake for an adult is 50–70 μg/day [181]. However, access to dietary selenium varies widely, from 7 μg/day to 5000 μg/day [189], depending on the presence of selenium in the local soil, local dietary characteristics, and accessibility. Selenium deficiency leads to bone disorders and stunted growth known as Kashin–Beck disease [183,190], while an excess of Se may lead to “garlic breath” [191] or, at higher intake levels, to alopecia and tooth darkening [192,193]. Some other sources of selenium in foods include derivatives of selenocysteine (**52**) and selenomethionine (**53**) in fruits and vegetables and the dimer selenocystine (**54**) in meats (Figure 18) [181]. These seleno amino acids can also be incorporated into proteins [194,195], providing alternative centers for redox activity [194,196].

Selenoneine was initially isolated and characterized spectroscopically as the dimer **55** from the blood and other tissues of the bluefin tuna, *Thunnus orientalis*; the monomer **12** was originally deemed too unstable to isolate [197,198]. Similar levels were found in mackerel, and 2- to 4-fold lower levels in tilapia blood, squid blood, chicken heart and liver, and porcine kidney. A reduction of **55** to the monomer was achieved with glutathione or dithiothreitol (DTT) [198]. Significantly stronger radical scavenging activity was demonstrated for **12** in the DPPH assay than **10** (1.9 μM vs. 1.7 mM, respectively) [197]. The alkaloid also reacted with methyl mercury and bound to heme proteins affording protection [198].

Clinical studies in 167 patients from four remote islands in Kagoshima Prefecture in Japan revealed high levels of **12** in their red blood cells, which were directly correlated with the frequency of fish consumption [199]. Another study examined **12** levels in red blood cells in the Inuit population from Nunavik in Northern Quebec, Canada, where high levels of **12** were also observed, together with the Se-methyl derivative, suggesting that **12** protects against the presence of MeHg in local fish [200,201]. In a more detailed study of **12** in fish muscles, the highest levels were observed in swordfish (2.8 nmol/g tissue), followed by bigeye, bluefin, and yellowfin tuna. Salmon, conger, saury, and sole had no **12** [202]. The Se:Hg molar ratio was also assessed, with swordfish having a ratio of 1 and sole a ratio of 217. An HPLC/MS-based system was developed to determine the concentrations of **12** and **10** in human red blood cells [203], and by using a pentabromobenzyl HPLC column, the monomers of **12** and **10** could be isolated from extracts of genetically modified yeast [204]. In 2014, a patent for the chiral total synthesis of **12** was described [181], and in 2019, an 11-step (2% overall yield) synthesis of **12** was presented, which allowed for biological activities to be identified, thereby distinguishing **10** and **12** [205]. Interestingly, and under physiological conditions, the reaction of the stable selenoneine diselenide occurred with several aromatic natural products, including resveratrol, vancomycin, and proansamitocin, to form derivatives through electrophilic aromatic substitution at positions *ortho* to phenolic groups [205]. This led to the suggestion that Se-derived adducts may be present at low levels in many natural matrices.

Selenoneine (**12**) has radical scavenging and strong antioxidant activity [181] in various models [206,207]. In addition, it ameliorates methylmercury accumulation in zebrafish in conjunction with the transporter OCTN1 [208], shows activity against the development of colorectal cancer in mice [209], inhibits tyrosinase in B16 melanoma cells and human melanocytes, probably by chelating copper at the active site of the enzyme [210], has ACE-inhibiting activity by binding to zinc [211], and crosses the blood–brain barrier only very slowly [212]. In order to account for the cytotoxicity in K562 human leukemic cells, it was proposed that **12** interacted with cysteine residues forming antioxidant, high molecular protein complexes [213]. Based on mammalian studies [198], **12** is regarded as probably non-toxic to humans. Further biological assessment of **12** appears to be limited by the absence of a commercial source [181].

### 4.2. Biosynthesis

**Figure 19:**
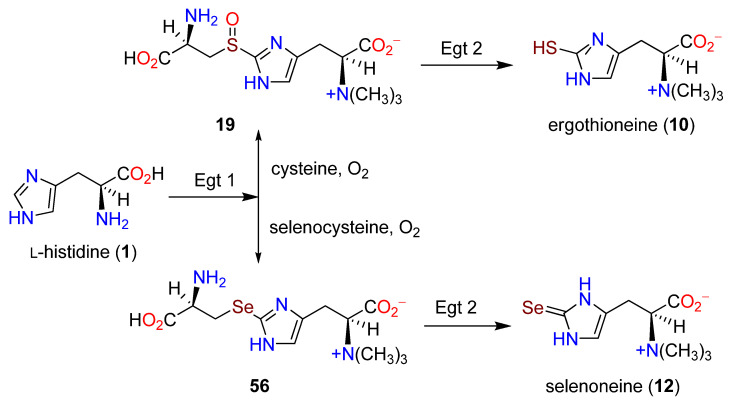
Divergent biosynthetic pathways from histidine (**1**) to ergothioneine (**10**) and selenoneine (**12**) through **19** and **56** (adapted from ref. [100]).

Aspects of the biosynthesis of **12** were discussed previously [100,169,170]. The addition of 10 μM Na_2_SeO_4_ was used in an EMM2 medium, and each stress change (e.g., limiting access to nitrogen or glucose) induced significant **12** production due to an increase in the transcription of the *egt1* gene. From the reaction of **10** and Na_2_SeO_4_, no **12** was produced, indicating a separation in the biosynthetic pathways and *not* an elemental replacement of Se for S in a pool of preformed **10** [100]. Overexpression of the *egt1* gene in *S. pombe* cultures raised the level to 1606.3 μM in the mutant strain from 0.3 μM in the wild-type strain. In a Δ*egt1* strain with added **10,** no **12** was formed. A double mutant of *egt1*^+^ and Δ*egt2* afforded a new intermediate, hercynylselenocysteine (**56**), and not the corresponding selenoxide, indicating a major difference in the function of Egt1 in the biosynthesis of **12**. A comparison of the two pathways is summarized in Figure 19 [100].

## 5. Conclusions

The histidine-derived alkaloids ergothioneine (**10**), ovothiol A (**11**), and selenothioneine (**12**) are exceptionally widely distributed at low levels throughout numerous phyla, including humans. They exhibit a range of biological activities, although their specific biological functions in situ are not well characterized beyond as “anti-oxidants”. Biosynthetically, their simplicity belies varied biosynthetic pathways, with three distinct approaches established for the formation of **10** and likely two for **11**. Much remains to be learned about these metabolites, particularly in terms of their dietary significance for humans.
